# Do associations between objectively-assessed physical activity and neighbourhood environment attributes vary by time of the day and day of the week? IPEN adult study

**DOI:** 10.1186/s12966-017-0493-z

**Published:** 2017-03-20

**Authors:** Ester Cerin, Josef Mitáš, Kelli L. Cain, Terry L. Conway, Marc A. Adams, Grant Schofield, Olga L. Sarmiento, Rodrigo Siqueira Reis, Jasper Schipperijn, Rachel Davey, Deborah Salvo, Rosario Orzanco-Garralda, Duncan J. Macfarlane, Ilse De Bourdeaudhuij, Neville Owen, James F. Sallis, Delfien Van Dyck

**Affiliations:** 1 Institute for Health and Ageing, Australian Catholic University, Level 6, 215 Spring Street, Melbourne, VIC 3000 Australia; 2 School of Public Health, The University of Hong Kong, Hong Kong, China; 3 Baker IDI Heart and Diabetes Institute, Melbourne, Australia; 4 Institute of Active Lifestyle, Faculty of Physical Culture, Palacký University, Olomouc, Czech Republic; 5 Department of Family Medicine and Public Health, University of California, San Diego, USA; 6 School of Nutrition and Health Promotion & Global Institute of Sustainability, Arizona State University, Phoenix, USA; 7 Faculty of Health and Environmental Sciences, AUT University, Auckland, New Zealand; 8 School of Medicine, Universidad de los Andes, Bogotá, Colombia; 9 Prevention Research Center, Brown School, Washington University in St. Louis, St. Louis, USA; 10 Graduate Program on Physical Education, Federal University of Parana, Curitiba, Brazil; 11 Department of Sports Science and Clinical Biomechanics, University of Southern Denmark, Odense, Denmark; 12 Centre for Research and Action in Public Health, Health Research Institute, University of Canberra, Canberra, Australia; 13 The University of Texas Health Science Center at Houston, School of Public Health in Austin, Austin, TX USA; 14 Center for Nutrition and Health Research, National Institute of Public Health of Mexico, Cuernavaca, Mexico; 15 Public University of Navarra, Navarra Pamplona, Spain; 16 Ghent University, Ghent, Belgium; 17 Swinburne University of Technology, Melbourne, Australia; 18 Research Foundation Flanders, Brussels, Belgium

**Keywords:** Built environment, Exercise, Accelerometry, Geographic Information Systems, International health

## Abstract

**Background:**

To more accurately quantify the potential impact of the neighbourhood environment on adults’ physical activity (PA), it is important to compare environment-PA associations between periods of the day or week when adults are more versus less likely to be in their neighbourhood and utilise its PA resources. We examined whether, among adults from 10 countries, associations between objectively-assessed neighbourhood environment attributes and moderate-to-vigorous physical activity (MVPA) varied by time of the day and day of the week. The secondary aim was to examine whether such associations varied by employment status, gender and city.

**Methods:**

This cross-sectional study included 6,712 adults from 14 cities across 10 countries with ≥1 day of valid accelerometer-assessed MVPA and complete information on socio-demographic and objectively-assessed environmental characteristics within 0.5 and 1 km street-network buffers around the home. Accelerometer measures (MVPA min/h) were created for six time periods from early morning until late evening/night, for weekdays and weekend days separately. Associations were estimated using generalized additive mixed models.

**Results:**

Time of the day, day of week, gender and employment status were significant moderators of environment-MVPA associations. Land use mix was positively associated with MVPA in women who were employed and in men irrespective of their employment status. The positive associations between MVPA and net residential density, intersection density and land use mix were stronger in the mornings of weekdays and the afternoon/evening periods of both weekdays and weekend days. Associations between number of parks and MVPA were stronger in the mornings and afternoon/evenings irrespective of day of the week. Public transport density showed consistent positive associations with MVPA during weekends, while stronger effects on weekdays were observed in the morning and early evenings.

**Conclusions:**

This study suggests that space and time constraints in adults’ daily activities are important factors that determine the impact of neighbourhood attributes on PA. Consideration of time-specific associations is important to better characterise the magnitude of the effects of the neighbourhood environment on PA. Future research will need to examine the contribution of built environment characteristics of areas surrounding other types of daily life centres (e.g., workplaces) to explaining adults’ PA at specific times of the day.

## Background

Ecological models of health behaviour posit that the neighbourhood built environment, along with intrapersonal, social environmental and policy factors, play an important role in shaping adults’ physical activity (PA) [1, 2]. Although PA can be influenced by the characteristics of places outside one’s neighbourhood (e.g., workplaces), a neighbourhood (defined as an area that surrounds someone’s home [3]) is a location of particular interest because it represents a universal daily life centre where people spend a significant share of their time and around which they organise their daily activities [4]. While everyone lives in a neighbourhood, not everyone spends time at a workplace or other daily life centres.

Many single-country studies have examined associations between aspects of the objective neighbourhood built environment and PA. These studies generally show that adults living in walkable neighbourhoods - characterized by high levels of residential density, interconnected streets, and high accessibility of shops, services and public transport - are more active than those living in low-walkable areas [5–8].

However, the conclusions drawn from single-country studies should be viewed with caution, as the variability of environmental attributes and PA in such studies is usually limited. This makes it difficult to accurately quantify the strength and shape of environment-PA associations [9, 10]. For example, while single-country studies have typically reported linear associations between residential density and walking for transportation [11–13], a recent investigation including comparable data from 14 countries observed a curvilinear relationship with both objectively-assessed and perceived measures of density [14, 15]. Specifically, residential density showed a positive association with walking for transport up to a certain level of density, and a negative association thereafter. These findings suggest that there are optimal levels of density that yield the best PA outcomes and increasing density in already dense environments may deter engagement in PA. Importantly, these findings emerged only after pooling the data from environmentally-diverse locations.

In examining pooled environment-PA associations based on multi-country data, it is particularly important to employ objective measures because there is some evidence that the level of potential bias in self-reported PA may vary across cultures and linguistic regions even when using (translated versions of) the same questionnaire [12]. The International Physical Activity and the Environment Network (IPEN) Adult study was the first multi-country cross-sectional study to estimate objectively-assessed environment-PA associations in 12 environmentally- and socially-diverse countries that used a comparable study design and similar methods [9]. Study findings showed that objectively-assessed residential density, intersection density, public transport density and number of parks were linearly positively related to accelerometer-based PA in all countries, and these associations were stronger than those previously identified by single-country studies [10].

In addition to examining the associations between objectively-assessed neighbourhood built environment characteristics and objectively-assessed PA accumulated ideally across the whole week, it is also important to consider whether these associations vary by time of the day and/or different days of the week [16]. This is because the extent to which the neighbourhood environment can potentially affect total PA depends on the amount of time a person spends in their neighbourhood [17]. For example, working adults are more likely to be in their residential neighbourhood during the weekend and in the early mornings, evenings and nights of weekdays (i.e., before and after work). For this reason, it is plausible to assume that associations between neighbourhood attributes and PA will be stronger for non-working periods of the day and week. Associations for these non-working periods should also be stronger than those related to total daily or weekly estimates of PA, which are routinely reported in the literature. This type of evidence would provide a more accurate and valid evaluation of the potential contribution of the neighbourhood environment to residents’ PA and, thus, strengthen the hypothesis of a causal relationship. The larger the differences in neighbourhood environment-PA associations (in the expected direction) between periods when adults are more vs. less likely to be in their neighbourhood and utilise neighbourhood resources, the greater the likely influence of neighbourhood-related factors on PA. Assuming positive associations between neighbourhood walkability and PA, a lack of differences in time-specific associations between the neighbourhood environment and adults’ PA across time periods would indicate that (1) residents of walkable environments (choose to) spend time in equally-walkable environments outside their neighbourhood; and/or (2) residents of walkable environments are more physically active than their counterparts irrespective of their location at a certain point in time. A lack of differences in time-specific associations would imply that resident self-selection into neighbourhoods (e.g., physically active people choosing to live in walkable areas) and individual predispositions to being physically active are the main factors underpinning a positive association between neighbourhood walkability and PA. Apart from helping address self-selection bias, time-specific analyses also assist the identification of time segments during which different socio-demographic groups (e.g., men vs. women) may be more responsive to potential environmental changes.

The main aim of the present analyses was to examine the extent to which associations between adults’ objectively-assessed neighbourhood environment attributes and accelerometer-assessed MVPA varied by time of the day and day of the week in 10 countries (14 cities). The secondary aim was to determine whether these moderating effects depended on geographical location (study city), gender and employment status.

## Methods

### Study design and neighbourhood selection

The IPEN Adult study was a multi-country cross-sectional epidemiologic study using a common design and comparable methods [9]. Participants were recruited from 17 cities across 12 countries: Australia (Adelaide, AUS), Belgium (Ghent, BEL), Brazil (Curitiba, BR), Colombia (Bogota, COL), Czech Republic (Olomouc and Hradec Králové, CZ), Denmark (Aarhus, DEN), Hong Kong/China (HK), Mexico (Cuernavaca, MEX), New Zealand (North Shore, Waitakere, Wellington, and Christchurch, NZ), Spain (Pamplona, SP), the United Kingdom (Stoke-on-Trent, UK), and the United States of America (Seattle/King County, Washington and Baltimore, Maryland region, USA). The current paper was restricted to 14 of the 17 study cities from 10 countries. Three cities were excluded because either no accelerometer data were collected (Adelaide, AUS) or no GIS data were available (Pamplona, SP; Hradec Králove, CZ).

The IPEN Adult study was designed to maximise variance in neighbourhood walkability and socioeconomic status (SES) by recruiting participants from neighbourhoods stratified by high/low walkability and high/low SES. Neighbourhood walkability index scores were created for small geographic areas in each city (“administrative units” roughly equivalent to US Census block groups) using Geographic Information Systems (GIS) data [18], with some differences by country [19]. Administrative units for each country that could be classified into one of the four neighbourhood types were selected. A balanced number of participants were then recruited from selected neighbourhoods [9].

### Participant recruitment

Households in the selected neighbourhoods were identified using databases from commercial and government sources in most study cities. In each selected household, an adult was invited to complete a survey and wear an accelerometer, with study dates ranging from 2002 to 2011 across countries. To account for seasonality effects on PA, seven of the 10 countries collected data across all seasons in a balanced manner. In contrast, Denmark and the UK collected data in Spring or Summer, when participants were more likely to engage in outdoor PA (including active transport) [9]. Brazil collected data in a single season (Spring) because Curitiba has relatively homogeneous average temperatures and humidity levels across the year. More information on participant recruitment can be found elsewhere [9]. Each country obtained ethical approval from their local institutional review boards, and all participants provided written informed consent.

### Participants

The entire IPEN Adult study consisted of 14,222 adults aged 18–66 years. Of these, 3,721 were excluded because the study site did not collect accelerometer data (Adelaide, AUS) or GIS data were unavailable (Pamplona, SP; Hradec Králove, CZ). About half of Hong Kong participants had no GIS data (*n* = 493) due to lack of resources for GIS data processing. About one-quarter of participants did not wear an accelerometer, either because they did not consent or the investigators could not afford collecting accelerometer data on all participants (*n* = 2,739). Participants who did not wear an accelerometer were more likely to be younger (*p* = 0.006), unemployed (*p* = 0.008) and without a college degree (*p* = 0.002). Of the remaining 7,269 participants, 360 did not provide valid accelerometer data, and 197 had missing data on socio-demographic and/or neighbourhood environment characteristics. This study examined 6,712 participants with ≥ 1 day of valid accelerometer data and with complete data on socio-demographic and objective neighbourhood environment characteristics. The socio-demographic characteristics of these participants by study city are reported in Table 1.

**Table 1:** Descriptive statistics of sample socio-demographic and accelerometer wear-time

	ALL CITIES	BEL	BRA	COL	CZE	DEN	HK	MEX	NZ				UK	USA	
Socio-demographics									City A	City B	City C	City D		City E	City F
N with ≥1 day valid PA data	6,712	1,040	331	107	252	273	257	625	426	448	463	425	147	1,213	705
Age, years *Mean (SD)*	43 (12)	43 (13)	42 (13)	44 (11)	37 (14)	40 (14)	43 (13)	42 (13)	42 (12)	42 (12)	40 (13)	42 (12)	43 (13)	44 (11)	47 (10)
Gender, *%men*	46.2	48.1	48.0	30.8	34.5	39.2	42.8	44.3	35.9	40.0	48.2	45.4	50.3	55.2	47.8
Education, *%*															
*Less than HS*	11.0	4.4	27.2	51.4	21.4	7.3	35.8	45.1	2.6	4.5	0.7	10.1	38.8	1.3	2.0
*HS graduate*	39.6	33.0	32.3	36.5	45.6	42.5	25.3	27.4	58.2	64.5	46.2	56.7	46.9	35.2	31.1
*College or more*	48.4	62.6	40.5	12.2	32.9	50.2	38.9	27.5	39.2	31.0	53.1	33.2	14.3	63.6	66.9
Employment status, *%employed - all*	79.2	80.8	79.8	64.5	76.2	75.5	63.0	72.2	78.2	84.6	87.0	82.4	61.9	81.5	83.5
*- among men*	85.5	85.0	88.7	90.9	85.1	75.7	75.5	88.8	86.3	88.3	88.3	84.5	67.6	87.1	85.5
*- among women*	73.8	76.9	71.5	52.7	71.5	75.3	53.7	58.9	73.6	82.2	85.8	80.6	56.2	74.5	81.8
Marital status, *%couple*	64.6	73.7	59.2	64.5	58.7	69.2	56.4	65.3	72.7	75.0	56.8	55.8	44.2	63.8	61.1
Accelerometer wear-time variables															
Valid weekdays *Mean (SD)*	4.7 (1.1)	4.9 (0.9)	4.9 (1.1)	5.0 (0.8)	4.2 (1.3)	5.1 (0.7)	4.1 (1.1)	4.2 (0.9)	4.3 (1.3)	4.4 (1.2)	4.5 (1.1)	4.5 (1.3)	4.5 (1.2)	5.0 (0.9)	5.1 (1.0)
Valid weekend days *Mean (SD)*	1.9 (0.5)	1.9 (0.4)	1.9 (0.5)	1.8 (0.6)	1.7 (0.5)	1.9 (0.3)	1.8 (0.5)	1.8 (0.6)	1.8 (0.6)	1.9 (0.7)	2.1 (0.7)	1.9 (0.6)	1.8 (0.4)	1.9 (0.4)	1.9 (0.5)
Valid hours / day *Mean (SD)*	14.3 (1.5)	14.7 (1.3)	13.7 (1.3)	13.8 (1.3)	13.6 (1.6)	14.9 (1.2)	14.3 (1.5)	13.9 (1.4)	14.0 (1.3)	14.0 (1.4)	13.9 (1.3)	13.8 (1.4)	14.3 (1.5)	14.5 (1.5)	14.6 (1.6)

*Notes:* City A: North Shore, B: Waitakere, C: Wellington, D: Christchurch, E: Seattle, F: Baltimore; *HS* High school, *MVPA* Moderate-to-vigorous physical activity, *SD* Standard deviation, *IQR* Interquartile range; valid days of accelerometer wear are those with 10+ valid hours of wear

### Measures

#### Socio-demographic characteristics

Self-reported socio-demographic variables included age, gender, education, employment status and marital status. As the classification of education varied by country, all data were categorized into ‘less than high school’, ‘high school’ and ‘college degree or higher’. Employment status was recoded as employed or not. Marital status was dichotomized into living as a couple versus not.

#### Objectively-assessed physical activity

MVPA was assessed objectively using accelerometers. Twelve cities used an ActiGraph device (Pensacola, Florida) and the four New Zealand cities used the Actical (Philips Respironics, Bend, Oregon). Data were collected by, or aggregated to, 1-min epochs. Non-wear time was defined as ≥60 min of consecutive zero counts. Participants were included in analyses if they had ≥ 1 valid wearing days containing ≥10 wearing hours. For ActiGraph data, Freedson cut points were used [20]. For Actical data, a new MVPA (≥730 cpm) cut point was developed to enable comparison with the ActiGraph-Freedson MVPA estimates [21]. Details on accelerometer data collection and reduction have been published elsewhere [11].

For the present analyses, data collected between 8:00 am and 11:59 pm were used. Data collected between midnight and 8 am were excluded because more than 50% of participants had 0 min of wear-time and over 70% had <30 min of wear-time per hour during this time period. For each participant, average minutes per hour of MVPA and monitor-wear time were computed on weekend days and weekdays for the following periods: 8:00 am to 8:59 am (early morning), 9:00 am to 11:59 am (morning), 12:00 pm to 1:59 pm (noon), 2:00 pm to 4:59 pm (afternoon), 5:00 pm to 7:59 pm (early evening), and 8:00 pm to 11:59 pm (late evening/night).

#### Objectively-assessed environmental characteristics

Objective measures of built environment attributes were developed by international teams using ArcGIS software (ESRI, Redlands, California) and a common set of GIS templates [19]. Neighbourhoods were defined by 0.5 km and 1.0 km street-network buffers around participants’ residential address using the “detailed no trim” setting to estimate accessible neighbourhood attributes. GIS templates were developed to ensure comparable GIS variables and document protocol adherence across teams. Detailed descriptions of the methods used to construct GIS variables and their comparability across study cities have been given elsewhere [19]. For the present analyses, the following variables computed for 0.5 km and 1.0 km street-network buffers were examined: net residential density; intersection density; land use mix (based on three land uses: residential, retail and civic); ratio of retail and civic land area to total buffer area; public transport density; and number of parks contained in or intersected by a buffer. In addition, we included the street-network distance from home to the nearest public transport stop.

### Data analytic plan

Descriptive statistics were computed for all variables by city and for the entire sample. Employment status distributions were also computed by gender to assist the interpretation of gender by employment status by city interaction effects (if any) examined in this study. To examine whether associations between objective neighbourhood environment variables and accelerometer-assessed MVPA (min/h) varied across time of the day and day of the week, generalized additive mixed models (GAMMs) were used [11, 22]. These are versatile regression methods that allow modelling of curvilinear relationships, positively skewed outcomes (e.g., minutes of MVPA), and correlated data (repeated observations from participants nested within administrative area units). GAMMs with Gamma variance and logarithmic link functions were estimated with random intercepts to account for clustering at the participant and neighbourhood levels. The Gamma variance and logarithmic link functions were the most appropriate (based on fit indices and analysis of residuals) to model positively skewed MVPA data with a standard deviation proportional to the mean. The antilogarithm of the regression coefficient estimates of these GAMMs represent proportional differences in the outcome associated with a 1-unit difference in a specific predictor.

Main-effect single-environmental-variable GAMMs estimated the dose-response relationships of all environmental attributes with MVPA, adjusting for study city, socio-demographic covariates, administrative-unit-level socio-economic status, time of the day (six periods modelled as 5 indicator variables), day of the week (weekend day vs. weekday), and accelerometer wear time. Curvilinear relationships of environmental attributes with MVPA were estimated using thin-plate spline smooth terms in GAMMs [22]. Smooth terms failing to provide sufficient evidence of a curvilinear relationship (≥5 difference in Akaike Information Criterion, AIC) were replaced by simpler linear terms. Appropriate two-way and three-way interaction terms were added to the main-effect GAMMs to examine whether associations of environmental attributes depended on the time of the day and whether the moderating effect of time of the day depended on the day of the week. Additional interaction terms were subsequently included to examine whether the moderating effects of time of the day and day of the week varied by gender, study city and employment status. Significance of interaction effects was evaluated by comparing AIC values of models with and without a specific interaction term (≥5 difference in AIC) [23]. Significant interaction effects were probed by computing associations at specific values of the significant moderator(s). A probability level of 0.05 was adopted, with all analyses conducted in R [24].

## Results

Table 2 reports descriptive statistics of the neighbourhood environmental attributes across cities. Between-city patterns of differences in these attributes have been described previously [17]. Table 3 reports descriptive statistics for MVPA by time period. In general, MVPA was lowest in the late evening/night period (8:00 pm to 11:59 pm) and highest in the afternoon (2:00 pm-4:59 pm) of weekend days (Table 3). Substantial variability in confounder-unadjusted estimates of MVPA at different time periods of the day was observed across cities. For example, Curitiba (BR) had higher, and the New Zealand cities had lower levels of MVPA in the mornings of both weekend days and weekdays. Bogota (COL), Olomouc (CZ), and Cuernavaca (MEX) had higher levels of MVPA in the mornings of weekdays but not in the mornings of weekend days (Table 3).

**Table 2:** Descriptive statistics of objectively-assessed environmental attributes (medians and interquartile ranges)

	ALL CITIES	BEL	BRA	COL	CZE	DEN	HK	MEX	NZ				UK	USA	
Environmental attribute									City A	City B	City C	City D		City E	City F
Net residential density (1000 / km^2^) – 1 km bf	2.4 (4.1)	5.2 (11.4)	4.7 (3.2)	9.8 (3.1)	18.9 (15.5)	4.8 (9.6)	65.5 (43.1)	2.2 (1.2)	1.7 (0.3)	2.1 (0.9)	2.5 (4.7)	1.5 (0.5)	4.2 (1.2)	2.2 (1.9)	1.5 (3.1)
Net residential density (1000 / km^2^) – 0.5 km bf	2.6 (4.2)	5.2 (8.3)	4.8 (3.4)	11.9 (6.1)	15.2 (17.9)	4.7 (14.9)	60.9 (56.0)	2.3 (1.9)	1.7 (0.4)	2.5 (1.0)	2.1 (4.0)	1.5 (0.7)	4.1 (1.1)	2.3 (2.0)	1.4 (3.3)
Intersection density (per km^2^) – 1 km bf	63 (55)	73 (59)	72 (22)	254 (135)	66 (25)	86 (26)	128 (89)	133 (55)	26 (9)	28 (6)	44 (19)	36 (8)	87 (38)	71 (29)	50 (27)
Intersection density (per km^2^) – 0.5 km bf	68 (67)	74 (73)	80 (26)	235 (176)	74 (28)	105 (39)	163 (93)	164 (113)	29 (19)	33 (16)	41 (31)	36 (13)	116 (49)	77 (39)	55 (36)
Land use mix (3 uses) – 1 km bf	0.46 (0.40)	0.60 (0.50)	0.58 (0.21)	0.57 (0.46)	0.59 (0.26)	0.91 (0.35)	0.88 (0.17)	0.46 (0.28)	0.37 (0.29)	0.42 (0.37)	0.53 (0.49)	0.29 (0.27)	0.20 (0.26)	0.38 (0.29)	0.39 (0.34)
Land use mix (3 uses) – 0.5 km bf	0.35 (0.54)	0.50 (0.54)	0.51 (0.33)	0.49 (0.42)	0.54 (0.29)	0.82 (0.44)	0.83 (0.24)	0.39 (0.49)	0.23 (0.45)	0.11 (0.45)	0.31 (0.62)	0.12 (0.24)	0.14 (0.37)	0.28 (0.49)	0.23 (0.48)
Ratio retail and civic land area to total bf area – 1 km bf^a^	0.09 (0.16)	0.09 (0.21)	0.13 (0.12)	0.08 (0.16)	0.04 (0.05)	0.44 (0.64)	0.59 (0.21)	0.16 (0.21)	0.07 (0.09)	0.07 (0.08)	0.13 (0.25)	0.06 (0.10)	0.02 (0.04)	0.06 (0.06)	0.08 (0.11)
Ratio retail and civic land area to total bf area – 0.5 km bf^a^	0.07 (0.17)	0.09 (0.23)	0.13 (0.13)	0.08 (0.13)	0.04 (0.04)	0.48 (0.90)	0.71 (0.46)	0.14 (0.26)	0.05 (0.16)	0.01 (0.09)	0.08 (0.22)	0.02 (0.07)	0.02 (0.06)	0.04 (0.10)	0.05 (0.13)
Public transport density – 1 km bf	14.3 (14.9)	7.6 (11.3)	25.4 (9.1)	0.9 (3.1)	14.5 (8.7)	9.1 (6.8)	13.0 (12.4)	25.6 (29.2)	19.7 (10.7)	7.2 (8.6)	15.1 (9.8)	16.3 (10.5)	24.0 (12.3)	15.7 (13.8)	13.5 (19.5)
Public transport density – 0.5 km bf	14.1 (19.7)	8.5 (10.9)	23.6 (15.5)	0.0 (2.3)	14.4 (13.4)	10.8 (10.4)	10.0 (21.3)	25.7 (48.6)	20.0 (15.9)	7.6 (9.9)	20.7 (13.3)	15.7 (17.3)	26.3 (15.9)	16.8 (22.0)	13.6 (26.0)
Street network distance to nearest transport stop (m)	239 (296)	258 (210)	162 (165)	1343 (2966)	233 (199)	234 (234)	353 (296)	229 (509)	190 (264)	297 (311)	149 (243)	238 (299)	194 (192)	225 (318)	264 (587)
No. parks contained or intersected by bf – 1 km bf	4.0 (5.0)	3.0 (5.0)	5.0 (6.0)	24.0 (19.0)	2.0 (4.0)	4.0 (4.0)	11.0 (14.0)	1.0 (2.0)	10.0 (7.0)	8.0 (5.0)	4.0 (4.0)	6.0 (3.0)	2.0 (2.2)	3.0 (5.0)	2.0 (2.0)
No. parks contained or intersected by bf – 0.5 km bf	1.0 (2.0)	1.0 (2.0)	1.0 (3.0)	7.0 (5.0)	1.0 (2.0)	1.0 (2.0)	3.0 (4.0)	0.0 (1.0)	4.0 (3.0)	3.0 (2.0)	1.0 (1.0)	1.0 (1.0)	1.0 (1.0)	1.0 (2.0)	1.0 (1.0)

*Notes:* City A: North Shore, B: Waitakere, C: Wellington, D: Christchurch, E: Seattle, F: Baltimore; bf = buffer

^a^ values truncated to 3.1 (1 value truncated for 1 km buffer measure and 22 for 0.5 km buffer measure). Since variables were positively skewed, medians and IQR are reported

**Table 3:** Accelerometry-assessed moderate-to-vigorous physical activity (min/h) by day of the week and time of the day

Day of the week	ALL CITIES	BEL	BRA	COL	CZE	DEN	HK	MEX	NZ				UK	USA	
Time of the day									City A	City B	City C	City D		City E	City F
Weekday															
8:00 am – 8:59 am	2.7 (4.4)	1.8 (2.6)	2.9 (5.2)	3.1 (4.8)	3.7 (4.8)	2.9 (4.7)	3.7 (5.4)	3.0 (5.3)	3.2 (5.1)	2.4 (3.9)	3.3 (4.5)	3.1 (5.0)	2.2 (3.6)	2.5 (4.5)	2.3 (3.8)
9:00 am – 11:59 am	2.8 (3.1)	2.4 (2.3)	2.2 (2.7)	2.9 (3.0)	3.2 (2.8)	2.5 (2.5)	3.7 (3.4)	2.8 (3.0)	3.5 (3.5)	2.9 (3.1)	3.8 (3.4)	3.6 (3.9)	3.5 (4.9)	2.4 (3.0)	2.0 (2.3)
12:00 pm – 1:59 pm	2.8 (3.0)	2.6 (2.4)	2.4 (2.8)	3.0 (2.9)	3.4 (3.2)	2.7 (2.7)	2.8 (2.7)	2.6 (2.8)	3.3 (3.3)	3.1 (3.1)	3.6 (3.4)	3.7 (3.9)	3.6 (4.7)	2.7 (2.9)	2.1 (2.6)
2:00 pm – 4:49 pm	2.8 (2.8)	2.5 (2.5)	2.5 (2.7)	2.9 (2.6)	3.9 (3.2)	2.9 (2.7)	3.1 (2.5)	2.3 (2.4)	3.3 (3.0)	2.9 (2.9)	3.6 (3.3)	3.6 (4.0)	3.3 (4.3)	2.4 (2.4)	1.9 (1.9)
5:00 pm – 7:59 pm	2.8 (2.8)	2.7 (2.6)	2.7 (2.6)	3.1 (2.7)	3.7 (3.3)	3.4 (2.8)	3.4 (3.1)	2.1 (2.4)	3.1 (2.8)	2.6 (2.7)	3.8 (3.3)	3.3 (3.5)	2.4 (2.8)	2.7 (2.8)	2.2 (2.4)
8:00 pm – 11:59 pm	1.6 (2.1)	1.6 (1.9)	1.4 (2.2)	1.5 (1.9)	2.2 (3.3)	1.8 (2.3)	2.4 (2.6)	1.4 (1.8)	1.3 (1.9)	1.3 (2.0)	1.9 (2.5)	1.5 (2.3)	1.3 (2.0)	1.6 (2.1)	1.4 (1.8)
Weekend day															
8:00 am – 8:59 am	2.2 (5.6)	1.3 (3.5)	2.9 (6.9)	2.2 (3.5)	1.8 (4.2)	1.0 (3.2)	3.0 (5.1)	2.0 (3.7)	3.5 (8.2)	2.2 (5.9)	2.5 (6.0)	2.6 (5.3)	3.1 (6.0)	2.5 (6.8)	1.9 (5.2)
9:00 am – 11:59 am	2.6 (4.0)	2.1 (3.3)	2.3 (3.3)	2.4 (3.7)	2.7 (3.6)	2.7 (4.1)	2.9 (3.5)	2.1 (3.3)	3.7 (5.1)	3.0 (3.9)	4.0 (4.8)	3.3 (4.7)	2.6 (3.4)	2.5 (4.1)	2.1 (3.8)
12:00 pm – 1:59 pm	3.1 (4.6)	3.0 (4.8)	2.2 (3.3)	2.8 (4.3)	3.2 (5.8)	3.0 (4.4)	3.2 (4.5)	2.3 (3.4)	4.1 (4.9)	3.5 (4.7)	5.1 (5.6)	3.6 (5.5)	2.9 (4.1)	3.1 (4.6)	2.3 (3.7)
2:00 pm – 4:49 pm	3.0 (4.0)	3.1 (4.3)	1.6 (2.4)	2.7 (3.6)	3.2 (4.6)	3.2 (4.1)	3.6 (4.5)	2.0 (2.9)	3.3 (3.6)	3.3 (4.1)	4.9 (5.2)	3.5 (4.3)	2.8 (3.7)	3.0 (4.0)	2.0 (2.7)
5:00 pm – 7:59 pm	2.4 (3.3)	2.7 (3.6)	1.6 (2.6)	2.5 (3.1)	3.1 (3.9)	2.5 (3.3)	3.2 (3.9)	1.5 (2.2)	2.6 (3.6)	2.0 (2.8)	3.3 (3.8)	2.5 (3.4)	1.9 (2.9)	2.4 (3.1)	1.8 (2.9)
8:00 pm – 11:59 pm	1.2 (2.0)	1.1 (1.8)	1.2 (2.2)	1.3 (2.0)	1.5 (2.7)	1.2 (2.2)	1.9 (2.2)	1.1 (1.8)	0.9 (1.7)	1.0 (1.7)	1.3 (2.3)	1.0 (2.2)	1.2 (2.1)	1.3 (2.0)	1.0 (2.0)

*Notes:* City A: North Shore, B: Waitakere, C: Wellington, D: Christchurch, E: Seattle, F: Baltimore

### Moderators of associations between objectively-assessed neighbourhood attributes and accelerometry-assessed MVPA

Table 4 reports significant moderators of associations between objectively-assessed environmental attributes and average MVPA (min/h). Time of day was a significant moderator of associations between MVPA and all environmental attributes with one exception. Specifically, street network distance to nearest public transport stop (km) (e^b^ = 1.00; 95% CI: 0.98, 1.02; *p* = 0.979) was unrelated to MVPA.

**Table 4:** Moderators of associations between objectively-assessed environmental attributes and accelerometry-assessed moderate-to-vigorous physical activity

Environmental attribute	Moderators (significant interaction effect)
Net residential density – 1 km bf	Gender x Time of the day Day of Week x Time of the day
Net residential density – 0.5 km bf	Gender x Time of the day Day of Week x Time of the day
Intersection density – 1 km bf	Day of Week x Time of the day
Intersection density – 0.5 km bf	Day of Week x Time of the day
Land use mix (3 uses) – 1 km bf	Gender x Employment status Gender x Time of the day
Land use mix (3 uses) – 0.5 km bf	Day of Week x Time of the day
Ratio retail and civic land area to total buffer area – 1 km bf	Day of Week x Time of the day
Ratio retail and civic land area to total buffer area – 0.5 km bf	Time of the day
Public transport density – 1 km bf	Day of Week x Time of the day
Public transport density – 0.5 km bf	Day of Week x Time of the day
Street network distance to nearest public transport stop	None
No. parks contained or intersected by buffer – 1 km bf	Time of the day
No. parks contained or intersected by buffer – 0.5 km bf	Time of the day

*Notes*: bf = buffer

Both net residential density measures within 0.5 km and 1 km buffers showed associations with MVPA that depended on time of the day, day of the week and gender (Table 5). Associations were consistently stronger for net residential density within 1 km buffers, whereby positive associations were observed across all periods of the day in men irrespective of the day of the week, and on weekdays irrespective of gender. Conversely, positive associations were observed only from 9:00 am onwards in women irrespective of the day of the week, and on weekend days irrespective of gender. On weekdays, associations tended to be stronger in the morning and from 5:00 pm onwards, while on weekend days they became stronger after 2:00 pm (afternoon, early evening and evening/night).

**Table 5:** Associations of objectively-assessed environmental attributes with accelerometry-assessed moderate-to-vigorous physical activity (min/h) by significant moderators

			Time of the day					
Environmental attribute	Day of the week	Gender	8:00 am – 8:59 am	9:00 am – 11:59 am	12:00 pm – 1:59 pm	2:00 pm – 4:59 pm	5:00 pm – 7:59 pm	8:00 pm – 11:59 pm
			e^b^ (95% CI) *p*-values	e^b^ (95% CI) *p*-values	e^b^ (95% CI) *p*-values	e^b^ (95% CI) *p*-values	e^b^ (95% CI) *p*-values	e^b^ (95% CI) *p*-values
Net residential density – 1 km bf (1000/km^2^)	All	Men	**1.007** ^**c**^ **(1.004, 1.011)** **<0.001**	**1.006** ^**c**^ **(1.003, 1.008)** **<0.001**	**1.002** ^**a**^ **(1.000, 1.005)** **0.033**	**1.004** ^**c**^ **(1.002, 1.006)** **<0.001**	**1.007** ^**c**^ **(1.004, 1.009)** **<0.001**	**1.007** ^**c**^ **(1.005, 1.010)** **<0.001**
	All	Women	1.003 (1.000, 1.006) 0.093	**1.003** ^**b**^ **(1.001, 1.005)** **0.008**	**1.003** ^**a**^ **(1.001, 1.006)** **0.016**	**1.005** ^**c**^ **(1.003, 1.008)** **<0.001**	**1.007** ^**c**^ **(1.004, 1.009)** **<0.001**	**1.005** ^**c**^ **(1.002, 1.007)** **<0.001**
	Weekday	All	**1.006** ^**c**^ **(1.004, 1.009)** **<0.001**	**1.005** ^**c**^ **(1.003, 1.007)** **<0.001**	**1.002** ^**a**^ **(1.000, 1.005)** **0.032**	**1.004** ^**c**^ **(1.002, 1.006)** **<0.001**	**1.006** ^**c**^ **(1.004, 1.008)** **<0.001**	**1.006** ^**c**^ **(1.004, 1.008)** **<0.001**
	Weekend	All	1.002 (0.998, 1.006) 0.272	**1.003** ^**a**^ **(1.000, 1.005)** **0.021**	**1.003** ^**a**^ **(1.000, 1.006)** **0.024**	**1.005** ^**c**^ **(1.002, 1.007)** **<0.001**	**1.007** ^**c**^ **(1.005, 1.010)** **<0.001**	**1.005** ^**c**^ **(1.003, 1.008)** **<0.001**
Net residential density – 0.5 km bf (1000/km^2^)	All	Men	1.003 (0.999, 1.007) 0.152	**1.003** ^**b**^ **(1.001, 1.005)** **0.007**	1.001 (0.999, 1.003) 0.289	**1.004** ^**b**^ **(1.001, 1.006)** **0.006**	**1.006** ^**c**^ **(1.003, 1.008)** **<0.001**	**1.005** ^**c**^ **(1.003, 1.008)** **<0.001**
	All	Women	0.998 (0.994, 1.001) 0.171	1.001 (0.999, 1.003) 0.321	1.002 (0.999, 1.004) 0.205	**1.004** ^**b**^ **(1.001, 1.007)** **0.003**	**1.006** ^**c**^ **(1.004, 1.008)** **<0.001**	**1.003** ^**b**^ **(1.001, 1.005)** **<0.001**
	Weekday	All	**1.005** ^**c**^ **(1.003, 1.007)** **<0.001**	**1.004** ^**c**^ **(1.002, 1.006)** **<0.001**	1.001 (1.000, 1.003) 0.139	**1.003** ^**c**^ **(1.001, 1.005)** **<0.001**	**1.005** ^**c**^ **(1.003, 1.006)** **<0.001**	**1.005** ^**c**^ **(1.003, 1.007)** **<0.001**
	Weekend	All	1.001 (0.997, 1.004) 0.772	1.002 (1.000, 1.003) 0.115	1.002 (0.999, 1.004) 0.217	**1.004** ^**c**^ **(1.002, 1.006)** **<0.001**	**1.006** ^**c**^ **(1.004, 1.008)** **<0.001**	**1.004** ^**c**^ **(1.002, 1.006)** **<0.001**
Intersection density – 1 km bf (100/km^2^)	Weekday	All	**1.760** ^**c**^ **(1.498, 2.068)** **<0.001**	**1.461** ^**c**^ **(1.257, 1.699)** **<0.001**	**1.225** ^**b**^ **(1.055, 1.423)** **0.008**	**1.180** ^**a**^ **(1.016, 1.370)** **0.030**	**1.314** ^**c**^ **(1.131, 1.526)** **<0.001**	**1.440** ^**c**^ **(1.240, 1.672)** **<0.001**
	Weekend	All	**1.352** ^**a**^ **(1.027, 1. 781)** **0.032**	**1.231** ^**a**^ **(1.005, 1.508)** **0.044**	**1.285** ^**a**^ **(1.053, 1.569)** **0.014**	**1.297** ^**b**^ **(1.064, 1.580)** **0.010**	**1.779** ^**c**^ **(1.460, 2.168)** **<0.001**	**1.952** ^**c**^ **(1.601, 2.379)** **<0.001**
Intersection density – 0.5 km bf (100/km^2^)	Weekday	All	**1.153** ^**c**^ **(1.097, 1.213)** **<0.001**	**1.109** ^**c**^ **(1.072, 1.148)** **<0.001**	**1.049** ^**b**^ **(1.014, 1.086)** **0.007**	**1.038** ^**a**^ **(1.006, 1.073)** **0.028**	**1.055** ^**b**^ **(1.020, 1.091)** **0.002**	**1.092** ^**c**^ **(1.055, 1.131)** **<0.001**
	Weekend	All	**1.274** ^**a**^ **(1.022, 1.589)** **0.031**	1.085 (0.920, 1.279) 0.334	1.078 (0.917, 1.268) 0.365	1.105 (0.941, 1.298) 0.222	**1.430** ^**c**^ **(1.218, 1.680)** **<0.001**	**1.605** ^**c**^ **(1.367, 1.886)** **<0.001**
Land use mix (3 land uses) – 1 km bf	All	Men	**2.101** ^**c**^ **(1.367, 3.228)** **<0.001**	1.100 (0.770, 1.573) 0.600	1.142 (0.803. 1.623) 0.460	1.311 (0.923, 1.861) 0.130	**2.060** ^**c**^ **(1.450, 2.924)** **<0.001**	**1.484** ^**a**^ **(1.044, 2.108)** **0.028**
	All	Women	0.841 (0.585, 1.209) 0.350	0.950 (0.706, 1.279) 0.736	1.024 (0.763, 1.374) 0.875	1.329 (0.991, 1.782) 0.057	**1.971** ^**c**^ **(1.470, 2.642)** **<0.001**	1.059 (0.790, 1.421) 0.700
Land use mix (3 land uses) – 0.5 km bf	Weekday	All	**1.583** ^**c**^ **(1.214, 2.063)** **<0.001**	1.082 (0.845, 1.385) 0.531	0.828 (0.647, 1.059) 0.132	1.091 (0.853, 1.394) 0.489	**1.713** ^**c**^ **(1.340, 2.190)** **<0.001**	**1.425** ^**b**^ **(1.115, 1.822)** **0.005**
	Weekend	All	0.737 (0.450, 1.204) 0.223	0.939 (0.673, 1.309) 0.709	1.111 (0.803, 1.537) 0.527	1.272 (0.921, 1.757) 0.144	**2.235** ^**c**^ **(1.619, 3.086)** **<0.001**	**1.392** ^**a**^ **(1.007, 1.924)** **0.045**
Ratio retail and civic land area to total bf area – 1 km bf	Weekday	All	No interaction by Time of the Day **1.237** ^**c**^ **(1.073, 1.426)** **<0.001**					
	Weekend	All	**0.630** ^**c**^ **(0.557, 0.733)** **<0.001**	1.061 (0.707, 1.594) 0.774	0.874 (0.586, 1.303) 0.507	**1.551** ^**a**^ **(1.042, 2.310)** **0.031**	1.283 (0.862, 1.910) 0.219	0.787 (0.525, 1.180) 0.246
Ratio retail and civic land area to total bf area – 0.5 km bf	All	All	No interaction by Time of the Day **0.982** ^**a**^ **(0.967, 0.998)** **0.028**					
Public transport density – 1 km bf (10/km^2^)	Weekday	All	**1.369** ^**c**^ **(1.287, 1.470)** **<0.001**	**1.157** ^**c**^ **(1.092, 1.226)** **<0.001**	**1.088** ^**b**^ **(1.027, 1.152)** **0.004**	**1.093** ^**b**^ **(1.032, 1.158)** **0.002**	**1.105** ^**c**^ **(1.043, 1.170)** **<0.001**	**1.088** ^**b**^ **(1.027, 1.152)** **0.004**
	Weekend	All	No interaction by Time of the Day **1.087** ^**c**^ **(1.047, 1.127)** **<0.001**					
Public transport density – 0.5 km bf (10/km^2^)	Weekday	All	**1.189** ^**c**^ **(1.136, 1.243)** **<0.001**	**1.099** ^**c**^ **(1.054, 1.146)** **<0.001**	1.042 (1.000, 1.086) 0.051	**1.051** ^**a**^ **(1.008, 1.095)** **0.019**	**1.063** ^**b**^ **(1.019, 1.107)** **0.004**	1.042 (1.000, 1.086) 0.051
	Weekend	All	No interaction by Time of the Day **1.059** ^**c**^ **(1.032, 1.086)** **<0.001**					
Street-network distance to nearest transport stop (1 km)	All	All	No interaction by Time of the Day 1.000 (0.979, 1.022) 0.979					
No. parks contained or intersected by bf – 1 km bf	All	All	**1.033** ^**c**^ **(1.020, 1.047)** **<0.001**	**1.030** ^**c**^ **(1.019, 1.041)** **<0.001**	**1.021** ^**c**^ **(1.010, 1.032)** **<0.001**	**1.024** ^**c**^ **(1.013, 1.036)** **<0.001**	**1.030** ^**c**^ **(1.018, 1.041)** **<0.001**	1.001 (0.991, 1.012) 0.787
No. parks contained or intersected by bf – 0.5 km bf	All	All	**1.098** ^**c**^ **(1.061, 1.137)** **<0.001**	**1.079** ^**c**^ **(1.048, 1.110)** **<0.001**	**1.041** ^**b**^ **(1.012, 1.071)** **0.006**	**1.043** ^**b**^ **(1.014, 1.074)** **0.004**	**1.057** ^**c**^ **(1.027, 1.088)** **<0.001**	0.993 (0.965, 1.022) 0.641

*Notes:* bf = buffer e^b^ = antilogarithm of regression coefficient; 95% CI = 95% confidence intervals. All analyses were adjusted for respondents’ age, gender, marital status, educational attainment, employment status, administrative-unit socio-economic status, study city, accelerometer wear time, time of the day, day of the week, and employment status by time of the day by time of the week interaction. e^b^ is to be interpreted as the proportional increase in physical activity associated with a 1-unit increase in the predictor. ^a^ *p* < .05; ^b^ *p* < .01; ^c^ *p* < .001. Significant effects in bold

Associations between MVPA and intersection density were stronger within 1 km than 0.5 km buffers. The associations with intersection density within 1 km buffers were all positive. However, their patterns across the day differed by the day of the week. Specifically, on weekend days, the associations were stronger in the early evening and late evening/night periods. In contrast, on weekdays, they were stronger in the early morning, morning and late evening/night periods (Table 5).

Gender and employment status moderated the association between MVPA and land use mix within 1 km buffers. While men showed a positive association irrespective of their employment status (e^b^ = 1.45; 95% CI: 1.23, 1.72; *p* < 0.001), women showed significant positive associations only if they reported being employed (e^b^ = 1.27; 95% CI: 1.08, 1.48; *p* = 0.003; for not employed: e^b^ = 0.92; 95% CI: 0.72, 1.18; *p* = 0.501). Further, a positive association was observed in women only in the early evening period, while in men the associations were also observed in the early morning and late evening/night periods. Day of the week, rather than gender, moderated the temporal patterns of associations between MVPA and land use mix within 0.5 km buffers. Positive associations were observed in the early evening and late evening/night periods on both weekends and weekdays. However, positive associations were present in the early morning only on weekdays.

Inconsistent patterns of associations were observed for the ratio of retail and civic land area to total area. A positive relationship (independent of time of the day) was found on weekdays for land use mix based on 1 km buffers, while a negative association was observed for the measure based on 0.5 km buffers irrespective of the day of the week. Inconsistent associations were also found across time of the day on weekend days for the measure based on 1 km buffers.

Both public transport density measures were consistently positively associated with MVPA on weekend days irrespective of the time of the day. However, the measure based on 1 km buffers yielded a stronger effect. On weekdays, the associations were strongest in the early morning and significant across the whole day but only for the measure based on 1 km buffers.

Number of parks in the neighbourhood was consistently positively related with MVPA across all periods up to 8:00 pm, irrespective of day of the week. However, for this characteristic, the associations were stronger when using a measure based on 0.5 km buffers, and strongest in the morning periods. All the above associations were linear and did not significantly vary by city.

## Discussion

The main aim of this study was to examine whether associations between objectively-assessed neighbourhood environmental characteristics and accelerometer-assessed MVPA differed across periods of the day and days of the week in adults from 14 cities across the globe. Five of seven environmental characteristics showed significant variations in associations across both time of the day and day of the week, while one characteristic showed significant variations in associations by time of the day only. Street-network distance to the nearest transport stop was the only environmental attribute with a stable non-significant association with MVPA, mirroring previous findings from the same study with respect to average daily minutes of MVPA [10].

On weekdays, stronger associations of net residential density, intersection density and land use mix with MVPA were observed in the morning periods and after 5 pm. Also, public transport density was most strongly related to MVPA in the mornings and early evenings (5 pm to 8 pm). These ‘before- and after-work’ time segments are weekday periods when adults are most likely to spend time in their residential neighbourhood. Other studies examining associations between the built environment and MVPA have identified these neighbourhood environmental attributes as the strongest correlates of MVPA, and especially of active transportation [25–27]. On weekdays, active transportation (e.g., walking to/from work, public transport points or doing errands) usually occurs in the morning and/or after 5 pm. Therefore, the time-specific associations observed in this study are logical. Similar relationships between neighbourhood walkability and moderate PA in the afternoon/early evening periods of working days were found in Sweden [16].

On weekend days, the associations of MVPA with net residential density, intersection density and land use mix were stronger in the afternoon/evening periods, while those with public transport density were positive and uniform across all periods of the day. This is in contrast to a Swedish study that found stronger associations between neighbourhood walkability and moderate PA from noon to 4 pm [16]. These between-study differences may be explained by differences in working-hours, climate, daylight patterns, PA preferences and retail trading hours. Swedish adults accrue a substantial proportion of daily PA through outdoor leisure [28–30], which is likely to take place during the warmer daylight periods of the day in the Scandinavian region. Also, shops and retail services typically open at 10 am and close at 2–4 pm on weekends in Sweden. In contrast, the typical weekend trading hours extend from 10 am to 6–10 pm in nearly all cities included in the present study. Thus, it is possible that, in our study, participants residing in more walkable areas engaged in within-neighbourhood active transport for shopping and social purposes in the afternoons and evenings of weekend days. The weaker environment-MVPA associations in the morning periods of weekend days than weekdays may be due to shops opening later on weekends and because a substantial proportion of those who work or study tend to extend their morning sleep by 0.5–2 h on weekends [31, 32].

Public transport density showed a positive association with MVPA on weekend days independent of the time of the day, while on weekdays the associations were stronger in the early mornings and evenings. Notably, the effects on weekend days were weaker than those in the early mornings and evenings of weekdays. These findings make sense because weekdays are usually associated with a more constrained activity schedule than are weekend days. Adults usually travel to/from work or school in the morning and late afternoon of weekdays, while on weekend days they have more freedom to determine their travel timing and options. Future research should examine the moderating effect of car ownership on this association given that a couple of recent studies found evidence of a positive effect of access to public transport on total PA in car owners only [33, 34].

It is interesting that the temporal patterns of associations between number of parks and MVPA did not depend on the day of the week. Positive associations were found until 8 pm, with stronger effects being observed in the mornings and early evenings. This could be explained by the fact that parks sometimes close in the evenings or may be perceived as less safe during late night hours or after the sun sets [35]. When people feel safer they tend to use parks more often and be more physically active there [36–39]. Present findings are consistent with prior reports that adults are more likely to engage in leisure-time PA in the mornings and late afternoons/early evenings [40]. In turn, these stronger associations in the mornings and early evening could guide future interventions in parks aimed at increasing park activity, such as community programs including PA and dancing classes similar to those implemented in Latin America [41].

The patterns of time-specific associations described above confirm that neighbourhood environmental factors play an important role in shaping adults’ PA. Notably, with the exception of dwelling density, the effect sizes for particular times of the day were 6–200% times greater than those related to average daily minutes of MVPA [10]. These are important findings as they provide estimates that are closer to the ‘true’ potential effects of the neighbourhood environment on PA. While important from a public health perspective, the commonly-reported associations between neighbourhood attributes and average daily or weekly PA represent ‘diluted’ approximations of neighbourhoods’ influences that depend on the spatial and temporal constraints experienced by the population studied (i.e., on their time budget and location of obligatory activities, such as workplace).

The present study revealed a few gender-specific findings. Net residential density and land use mix were correlated with MVPA in the morning periods in men but not in women. Although employed women showed a positive association between land use mix and MVPA, unemployed women did not. Some women may choose not to work or may not be able to afford working (high childcare costs) in order to care for their young children and, for the same reason, not regularly engage in active transport for shopping/errand purposes with their children [42]. In this regard, higher levels of physical fatigue experienced by mothers of young children and the psycho-social pressures of managing the demands of children who get fatigued from walking long distances have been identified as barriers to engaging in active transport in mothers [42]. Some working women with children may prefer using motorized rather than active transport to/from work for safety issues [43] or because they need to drop and pick up their children from childcare [44]. After work, they may share childcare with their partner and be able to walk to/from local shops and services in more-walkable neighbourhoods. In support of this contention, employed fathers with employed wives have been found to average three to six more weekly hours of solo childcare than fathers with non-employed wives [45].

Some of the patterns of associations observed in this study were similar to, and others differed from, those observed in relation to overall weekly accelerometer-assessed MVPA in the same sample [10]. Both investigations revealed stronger associations with measures of net residential density, intersection density and public transport density based on 1 km buffers, and with number of parks within 0.5 km buffers. However, the present investigation also revealed time-specific associations with land use mix and retail and civic land use ratio which were not identified as correlates of overall accelerometer-assessed MVPA. This is most likely due to the greater level of contextual precision (i.e., greater ability to capture/isolate time periods spent in the neighbourhood) of the present analyses and the superior statistical power associated with having multiple measures of MVPA per day of the week for each participant.

It is also important to note that, while temporal patterns of MVPA differed across cities, environment-MVPA associations and the moderating effects of gender and employment status on these associations did not. These findings provide support for the generalizability of the potential impact of the neighbourhood built environment on adults’ time-specific PA across countries and continents [10, 11, 14, 15, 46, 47].

### Implications of findings

The present findings have important implications for future studies, policy and practice. The fact that time-specific environment-MVPA associations were found for most environmental characteristics in all participating cities indicates that it is important to consider the temporal patterns of such associations in future analyses and interventions. While common practice in studies on the effects of the school environment on PA [48], most research on the neighbourhood environment has not adopted a time-specific approach, possibly because there is more inter-individual variability in the timing and amount of time adults spend in their neighbourhood.

As expected, associations tended to be stronger for periods of the day or week when adults were likely to be in their neighbourhoods (and awake). This pattern of results provides stronger support for a causal interpretation. If the neighbourhood environment truly influences one’s MVPA, it is expected to influence it the most when the person is in the neighbourhood. Two environmental attributes (land use mix and retail and civic land use ratio) showed significant associations with MVPA only at certain times of the day, while they failed to contribute to the explanation of overall MVPA [10]. These findings suggest that to increase total MVPA in adults, it remains important to conduct multi-dimensional, multi-level interventions, focusing on appropriate neighbourhood environmental changes as well as other strategies and settings, such as interventions in the workplace. Multi-level approaches are expected to lead to cumulative increases in PA and larger health effects [49–51]. These findings also highlight the importance for this research field to consider space and time constraints that limit one’s ability to engage in PA and utilise PA resources in their environment. The latter observation applies to the study of neighbourhoods as well as other daily life centres (if any). In particular, future studies would need to examine the role of environmental attributes around adults’ workplaces to establish their contribution to explaining PA during working hours (e.g., 9 am - 5 pm) on weekdays. Finally, future studies should also make use of Global Positioning System (GPS) monitors to help characterise the temporal patterns and quantify the time adults spend in different daily life centres. This would enhance our understanding of the contribution of the environmental characteristics of residential neighbourhoods versus other daily life centres (e.g., workplaces) to adults’ PA [52].

Lastly, while environmental initiatives to increase PA seem to have a similar potential across diverse countries, the gender- and employment status-specificity of a few of the current findings suggests that the effectiveness of environmental changes may vary across socio-demographic groups. In this study, positive associations between land use mix and MVPA were more consistent in men than women. Women typically experience more space and time constraints than men due to their greater domestic responsibilities [44, 45], which are bound to limit their ability to capitalize on neighbourhood opportunities to be physically active.

### Study strengths and limitations

Several study strengths and limitations should be acknowledged. Strengths included that this was a multi-country study with a large sample, standardized protocols and measures, using appropriate pooled analyses maximizing the statistical power. Both objective GIS and MVPA data were collected, which is a unique study feature on such a large scale. The unavailability of GPS data is a study limitation precluding the linkage of time-stamped MVPA data with specific geographical locations (inside or outside the residential neighbourhood). However, analyses of additional data collected on samples from Mexico, Colombia and Brazil showed that parks and streets were the main places where participants were active, and the use of public places was significantly associated with MVPA [53]. The cross-sectional study design does not permit an assessment of causal effects. Another limitation was the inability to examine associations with specific types of PA (e.g., walking, occupational PA or engagement in sport) which cannot be inferred from accelerometry data. Finally, it would have been useful to increase the accuracy of the estimated associations by adjusting for climatic conditions (e.g., temperature and rainfall) at the time of MVPA assessments. However, climatic data were unavailable.

## Conclusions

In conclusion, the present study provided a comprehensive attempt to relate objectively-assessed neighbourhood environmental attributes with the timing of MVPA across the day and across days of the week. To better understand the impact of the neighbourhood environment on adults’ PA and develop effective environmental interventions, it is important to identify and examine the periods during which residents can potentially capitalise on the PA opportunities provided by their communities. This requires the identification of time and space constraints of obligatory or semi-obligatory activities in key socio-demographic groups (men and women; employed and unemployed).

## Abbreviations

AIC: Akaike Information Criterion
AUS: Australia
BEL: Belgium
BR: Brazil
CI: confidence intervals
COL: Colombia
cpm: Counts per minute
CZ: Czech Republic
DEN: Denmark
GAMMs: Generalised additive mixed models
GIS: Geographic Information Systems
GPS: Global Positioning System
HK: Hong Kong
IPEN: International Physical Activity and the Environment Network
MEX: Mexico
MVPA: Moderate-to-vigorous physical activity
NZ: New Zealand
PA: Physical activity
SES: Socioeconomic status
SP: Spain
UK: United Kingdom
USA: United States of America
